# Glass–Zirconia Composites as Seals for Solid Oxide Cells: Preparation, Properties, and Stability over Repeated Thermal Cycles

**DOI:** 10.3390/ma16041634

**Published:** 2023-02-15

**Authors:** Magdalena Kosiorek, Agnieszka Żurawska, Leszek Ajdys, Anna Kolasa, Yevgeniy Naumovich, Paulina Wiecińska, Aleksey Yaremchenko, Jakub Kupecki

**Affiliations:** 1 Institute of Power Engineering, Mory 8, 01-330 Warsaw, Poland; 2 Center for Hydrogen Technologies (CTH2), Institute of Power Engineering, Augustowka 36, 02-981 Warsaw, Poland; 3 Institute of Heat Engineering, Warsaw University of Technology, Nowowiejska 21/25, 00-665 Warsaw, Poland; 4 Faculty of Chemistry, Warsaw University of Technology, Noakowskiego 3, 00-664 Warsaw, Poland; 5 CICECO-Aveiro Institute of Materials, Department of Materials and Ceramic Engineering, University of Aveiro, 3810-193 Aveiro, Portugal

**Keywords:** SOE, SOFC, alumino-borosilicate glass, composite seal, YSZ

## Abstract

This study focuses on the preparation and characterization of composite gaskets designed for the sealing of the solid oxide cell stacks operating below 700 °C. The seals were fabricated with the addition of various amounts (10–90 wt.%) of 3 mol.% yttria partially stabilized zirconia to a BaO-Al_2_O_3_-CaO-SiO_2_ glass matrix. The sample gaskets in the form of thin frames were shaped by tape casting. The quality of the junctions between the composites and Crofer 22APU steel commonly used as an SOC interconnect was evaluated after thermal treatment of heating to 710 °C, then cooling to the working temperature of around 620 °C and then leaving them for 10h in an air atmosphere, before cooling to room temperature. The samples were also studied after 3, 5, and 10 thermal cycles to determine the changes in microstructure and to evaluate the porosity and possible crystallization of the glass phase. The compression of the seals was calculated on the basis of differences in thickness before and after thermal treatment. The influence of zirconia additions on the mechanical properties of the seals was studied. The experimental results confirmed that glass–ceramic composites are promising materials for gaskets in SOC stacks. The most beneficial properties were obtained for a composite containing 40 wt.% of YSZ.

## 1. Introduction

Solid oxide cells (SOC) are among the most promising energy-related technologies currently being developed by scientists around the world. They provide an efficient solution for the generation of electricity in fuel cell mode (SOFC) and effective means of hydrogen production when operated as solid oxide electrolyzers (SOE) [1,2,3,4].

One key issue in SOC technology relates to the reliability of seals—to ensure electric insulation and gas tightness—in the demanding conditions found in stacks. There are various approaches to SOC stack design, but planar cells show many advantages such as manufacturing simplicity, high power density, fuel flexibility, and profitability [5,6]. Therefore, the seal is usually applied as a thin planar gasket. The main role of the seal is to prevent the fuel and oxidant from mixing, creating a barrier between the cathode and anode sides of the following compartments and thus ensuring the conditions for the proper long-term operation on the cells [7,8,9].

There are several conditions that must be fulfilled to ensure reliable seals in a SOC stack. They must demonstrate no gas (incl. H_2_) permeability in the relevant temperature range (600–700 °C for the current design of intermediate temperature stacks of SOCs, with several stack designs operating in the range from 750 to 850 °C, above which they are considered as a previous generation mostly relying on electrolyte supported cells) and stability with regard to oxidizing and reducing atmospheres. They have to be able to withstand thermal cycling over the lifetime of the stack, which is estimated in the tens of thousands of hours. With rigid-type seals, the value of the thermal expansion coefficient (TEC) of the seal must be similar to the values of the stack components in order to prevent thermal stress fractures. Seals also have to act as an electrical insulator, that is chemically compatible with other components of the stack. In terms of economics, a seal must be easy to shape and process and relatively inexpensive in order to enable upscaling of production [10]. Proper sealing is crucial for good stack operation. Any failed seal causing leakage significantly impacts efficiency and increases the risk of overheating [11], even if control algorithms go some way toward maintaining a SOC stack that has experienced a failure of sealing in operation conditions [12].

Seals for SOC stacks can be categorized into three types: (i) compressive seals, (ii) compliant seals, and (iii) rigidly bonded seals. Rigidly bonded seals have many advantages over the other two and are preferred for the application described in this paper. Recent studies have shown that glass-based seals are typically chosen for use in SOC stacks as their properties can be tailored to meet the requirements listed above. Such glasses consist of a network former, a network modifier, intermediate oxides, and an additive [10,12,13]. The components are modified to influence thermal, chemical, mechanical, and electrical properties. Two approaches may be adopted when altering the composition of the seal: ceramic powder can be added to the glass matrix as a filler or reinforcement during glass synthesis or composite formation. The glass matrix which is most commonly applied in SOC technology is based on the BaO–CaO–SiO_2_ system (BCS), BaO-Al_2_O_3_-CaO-SiO_2_ system (BCAS), or BaO-Al_2_O_3_-B_2_O_3_-SiO_2_ (BAS) modified with B_2_O_3_ [14,15,16]. A seal of this type becomes a glass ceramic due to crystallization during thermal treatment. It has been found that the crystallization of the glass can be advantageous because the resulting material is typically stronger than at the start of the process [16]. However, because the final joint is brittle, it might fracture when exposed to tensile stresses due to thermal expansion mismatches between the sealant and adjacent substrates. On the other hand, a composite seal, in which a crystalline ceramic phase is dispersed uniformly in a glass–ceramic matrix, seems to be more beneficial, as it is easier to tailor parameters such as viscosity or TEC by changing the volume content and particle size of the reinforcing chosen ceramic phase. Such composites also have a self-healing character, as the glass matrix remains viscous. Many different types of reinforcement have been studied to date, including: Al_2_O_3_ [17,18,19], ZnO [20,21], Nb_2_O_5_ [22], V_2_O_5_ [21], AlN [23], Y_2_O_3_ [24], Cr_2_O_3_ [25], MgO [26,27], Bi_2_O_3_ [28,29], BaO [30], La_2_O_3_ [31], mica [32,33], and ZrO_2_. ZrO_2_ reinforcement is the most interesting one for the authors because it is seen as an additive stimulating crystallization of the seal, which is often desirable for enhancing the mechanical properties of the joint, depending on the SOC stack design. Its TEC value is often very close to the TEC value of the chosen glass matrix, as it is expected to join the yttria-stabilized zirconia (YSZ) electrolyte of the cell to the metallic interconnect. Malzbender and Zhao studied partially crystallized and almost fully crystallized seals based on a BaO–CaO–SiO_2_ glass matrix [34]. For the partially crystallized case, YSZ was added as a reinforcement component. The composite was screen-printed on Crofer 22APU steel. The results showed no considerable change in the strength for the almost fully crystallized sealant, while a severe decrease in the strength caused by viscous deformation was observed for the partially crystallized sealant with YSZ powder reinforcement. As a side note, since the YSZ particles were big grains (~5–20 µm), they did not affect the glass nucleation and crystallization. Gross et al. came to similar conclusions in their study on composite materials, also based on a BCS glass matrix and various filler materials, such as YSZ fibers or particles and silver particles [35]. In order to determine the influence of the filler material on the composite, tensile strength tests were carried out on the joints with Crofer 22 H steel. The composite with YSZ particles achieved the lowest measured values. The YSZ particles used were of the technical kind, and the powder had a median particle size of 15 μm. In another report, the same researchers concluded that additions of 20 wt.% YSZ to BCS glass were optimal with respect to the desired thickness of the seal of 250 μm [36]. Adding greater amounts of filler material to the glass matrix changed the crystallization process dramatically, with low crystallization rates observed. Moreover, it was found that the additions of YSZ resulted in only a minor increase in the softening temperature of the sealant. The stack sealed with the said composite was tested for 1000 h at 800 °C, maintaining cell performance during five thermal cycles. The same composite was used for the effective sealing of the stack, which was subjected to continuous operation for 70,000 h at 700 °C, as reported by Blum et al. [37]. In contrast, Heydari et al. investigated the influence of nano-zirconia powder as a reinforcement to a BCAS glass matrix [38]. The mechanical properties of the specimens heat-treated at 800 °C for different times were determined. The growth of crystalline phases was seen with a longer treatment time. The microhardness of the specimens increased with the increasing amounts of zirconia, but an increase in the heat treatment time did not affect it. By raising the amount of zirconia nanoparticles to 10 vol.%, the flexure strength of the specimens also increased. Another research campaign on a glass seal reinforced with YSZ was carried out with the use of a BaO-B_2_O_3_-SiO_2_-ZnO-MgO glass matrix and TZ-8YS zirconia powder from the Tosoh Company with the mean grain size of 0.5 µm [39]. In experiments, the glass seal with the addition of 20 wt.% YSZ exhibited a very low leakage rate of 0.01 sccm cm^−1^ and leakage rates below 0.02 sccm cm^−1^ were obtained after ten thermal cycles between 750 °C and 300 °C under a compressive load of 0.2 MPa. Further studies [40] demonstrated that an 8YSZ-(MgO-Al_2_O_3_-SiO_2_-BaO-B_2_O_3_-SrO) glass composite has relatively low devitrification rates at an SOC operating temperature (750 °C). 

Many studies concerning glass–ceramic composites report the properties of sealing glass and the influence of ceramic powder addition to the glass matrix at operating temperatures of around 800 °C. However, the current focus in SOC development is to lower the operating temperature below 700 °C using, for example, Ni/YSZ-supported cells with an oxygen electrode made of (La_0.6_Sr_0.4_)_0.99_CoO_3_ [41]. Such electrode materials should not operate above 750 °C due to rapid degradation. Therefore, the stability of the seals needs to be studied at the relevant temperatures. Preferably, the sealing temperature of a stack of this type should be below 750 °C, which means a relatively low softening temperature of the glass matrix. Therefore, the aim of the present work was to study the influence of YSZ reinforcement on a commercially available glass based on the B_2_O_3_-modified BCAS system, designed for sealing of SOC operating in temperatures < 700 °C. The motivation was to obtain a composite gasket characterized by high gas tightness, low porosity, resilience to repeated thermal cycles, good junction with the other components, and low manufacturing costs. The important and innovative objective of the study was to design a composite gasket with a desired compression rate compared to the glass gasket without reinforcement using easily accessible materials and methods of preparations, which can be upscaled to the mass-production level. 

## 2. Materials and Methods

### 2.1. Materials and Preparation

The study focused on the development of composite sealant for SOC stack application. The key material was Schott glass powder GM31107 from the BaO-Al_2_O_3_-CaO-SiO_2_ system modified with B_2_O_3_ [42]. The filler material was 3 mol% yttria partially stabilized zirconia (YSZ), trade name YSZ–714 from ABSCO Materials. The powders underwent a series of analyses to characterize their properties in detail. A Zetasizer Nano ZS Malvern Panalytical was used to measure the particle size. Scanning electron microscopy (SEM) was used to determine the morphology of the powders. High-temperature microscopy (Carl Zeiss Jena, MHO-2) was used to compare the sintering behavior of the glass and mixed powders. The samples were prepared by mixing the glass powder or a mixture of powders with a small amount (<1 wt.%) of PVA (polyvinyl alcohol), a commonly used binder, to improve wettability for the purpose of uniaxial pressing. The final shape of the sample was a cylinder with a diameter of 2 mm and a height of 2.5 mm, pressed under 50 MPa. The characteristic temperatures of composites consisting of 5–30 wt.% of YSZ in the glass matrix were estimated on the basis of the obtained images. Sintering temperature is defined as the temperature at which the grains begin to soften at the border of their contact, with a simultaneous reduction in dimensions without changing its initial form (the first point was marked below 98% of the original sample size with sharp edges). Softening temperature is pointed where the first sign of softening of the shaped body is observed, found on the basis of a change in the surface or rounding or bending of the fitting. Hemisphere temperature is marked where the height of the sample is equal to 1/2 of the base of the shape [13].

**Figure 1:**
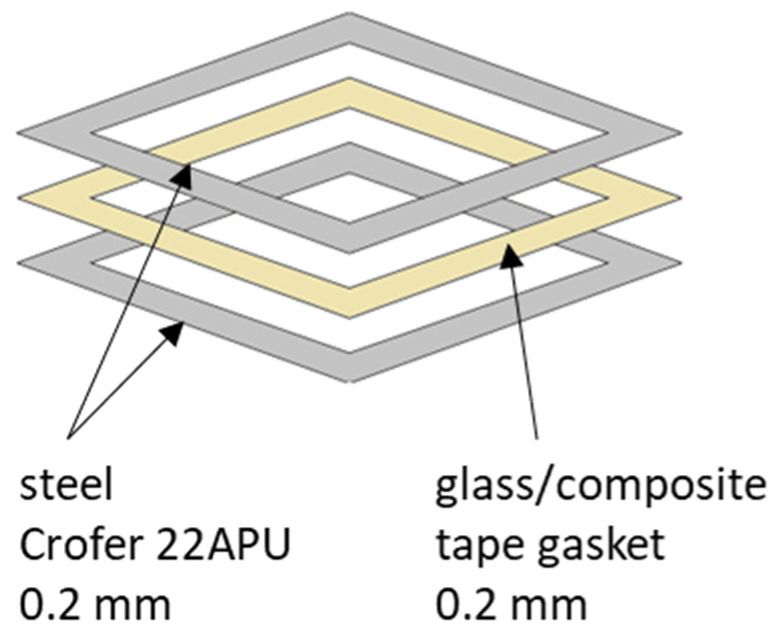
Diagram of a sample in the form of a composite frame sandwiched between Crofer 22APU steel.

X-ray diffraction (XRD) was used to determine the phase composition of the glass powder in the as-received form. After characterization of the powders, composites containing 10–90 wt.% YSZ in the glass matrix were fabricated by tape casting. A reference sample was made of glass alone. The reference slurry was composed of 29 wt.% solvent (ethanol), 1.2 wt.% dispersant (KD-2, Croda PLC, Snaith, UK), 1.8 wt.% binder (polyvinyl butyral, ABCR GmbH, Karlsruhe, Germany), 2.0 wt.% plasticizer (dibutyl phthalate, Aldrich/Merck KGaA, Darmstadt, Germany), and 66 wt.% of the powders. The desired homogeneity was reached by milling in a planetary ball mill (Pulverisette 6, Fritsch GmbH, Weimar, Germany) with a speed of 150 rpm for at least 90 min. Zirconia balls of 5 mm diameter were used in a 1:2 weight ratio to the slurry. The slurry was then cast at room temperature on the polyvinyl foil surface using a tape caster (MSK-AFA-II, MTI Corporation, Richmond, CA, USA) with a speed of 1 mm/s. The doctor blade was calibrated in order to obtain thin tapes with a final thickness of 0.2 mm. After drying at room temperature for a minimum of 12 h, the films were cut into frames with a laser plotter and put into steel frames made from Crofer 22APU. Figure 1. shows a sandwich sample in diagrammatic form. 

### 2.2. Characterization of Composites

Prepared sandwich samples were placed in the specially designed holder mounted in the furnace and then heated while remaining in a mechanically compressed state under pressure of 0.38 MPa, which corresponds to ca. 5 kg per square centimeter in accordance with the heating procedure for an average SOC stack. The sample was initially heated to the furnace setpoint 710 °C, which is above the sealing temperature of the glass. The dwell time for this step was 2 h in order to make sure that the material was evenly distributed and softened in the whole volume. Afterwards, the temperature was decreased to the desired operating point of 630 °C, and after 10 h of dwell in these conditions, the furnace was cooled to room temperature. The thickness of the samples was measured before and after the heating procedure. The thickness change after heating, given as the % of the thickness of the raw tape used for assembly of the sandwich sample, can be interpreted as the compression rate of the gasket, which reveals the shrinkage of the material in the *z*-axis.

$$CR=\left(1-\frac{L_{sah}}{L_{rt}}\right)\times 100\%$$

where *CR*—compression rate

$L_{sah}$—thickness of a seal after heating

$L_{rt}$—thickness of the raw tape

The next step was to prepare the cross-sections of the heated sandwich samples for SEM analysis in order to determine the quality of the sealing material joining the steel. In this case, the samples were mounted in epoxy, cut into pieces, and then ground (using fine sanding paper graded from 180 to 2500 grain) and polished (using 1 µm diamond paste). Moreover, to verify the applicability of a seal, selected samples were investigated over 3, 5, and 10 thermal cycles. For this reason, the new sandwich samples were prepared by assembling seal tape (circle with a diameter of 10 mm cut with laser plotter) placed between two Crofer 22APU steel coupons with a diameter of 10 mm and heated under pressure in accordance with the previously described procedure. The new set of samples was then ground and polished for the purpose of carrying out SEM analysis of the cross-sections. SEM/EDS analysis was carried out with a Zeiss ULTRA plus FESEM and JEOL JSM-6010PLUS/LA InTouchScope. In order to estimate the value of the porosity of both reference and composite sealant layers after the sealing procedure, SEM images of cross-sections of the samples were analyzed using the open-access program ImageJ [43], which allows for definition and measurement of the area of the visible pores, observable on cross-sections [44,45]. A pore was defined as a pseudo-particle with an observable area of at least 0.04 μm^2^ (a particle with at least 4 pixels in available resolution); this allowed for avoidance of an impact from the dithering noise. Four to six SEM images with a magnification of 1000…3000 times were analyzed for each material. After analysis, the collected data sets were used to estimate the average values of the visible porosity and standard errors with a 95% confidence interval.

In order to verify the phase composition and crystal structure of the obtained materials, X-ray diffraction (XRD) measurements were carried out in a range of 2θ = 10–110° with step 0.013° on a Panalytical Empyrean diffractometer (with Cu Kα radiation) equipped with a PIXcel3D detector. The samples for XRD analysis were prepared by tape casting, cut, stacked on the steel plates, and then heated according to the previously described procedure. The glass and composite samples were crushed and ground using a pestle and mortar in order to obtain powder samples.

## 3. Results and Discussion

### 3.1. Starting Materials and Formation of the Composite

**Figure 2:**
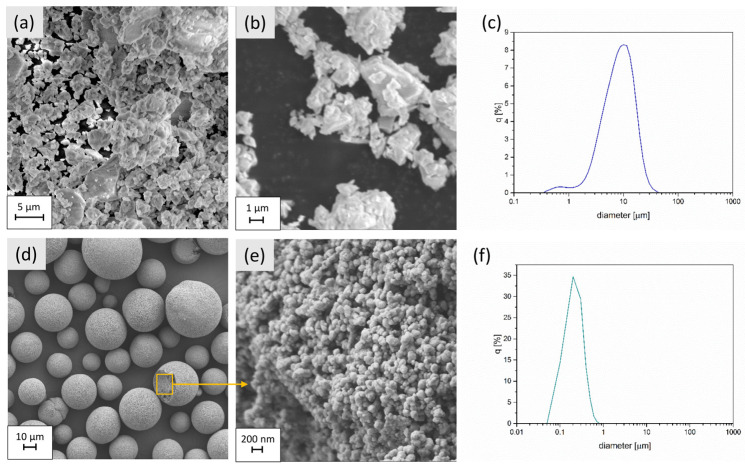
Microstructure and histogram of the grain size distribution of glass powder Schott GM31107 (**a**–**c**) and YSZ powder (**d**–**f**).

Figure 2a,b. presents an SEM image of the glass powder. The particles are irregular in shape and of various sizes. Large particles with a length of about 8 μm as well as finer ones in the range of 1–4 μm can be seen. The powder is agglomerated. Figure 2d,e shows the micrographs of YSZ powder. The as-received material is in granulated form. The results indicate that the powder granules are of different sizes, and their diameters are in the range of 10–85 μm. The particles that make up the granules are characterized by an irregular, globular shape, and their diameter does not exceed 200 nm.

The particle size distribution of both powders is shown in Figure 2c,f. The histogram made for the glass powder indicates a definite peak, but the range of diameter values is quite wide, as was proven by the SEM images. The mean size of the grains was 8.8 µm. The distribution range of the particle size for YSZ powder was narrower; the mean size of the particles was around 0.4 µm, whereas the average glass powder particle size was some 20 times greater.

**Figure 3:**
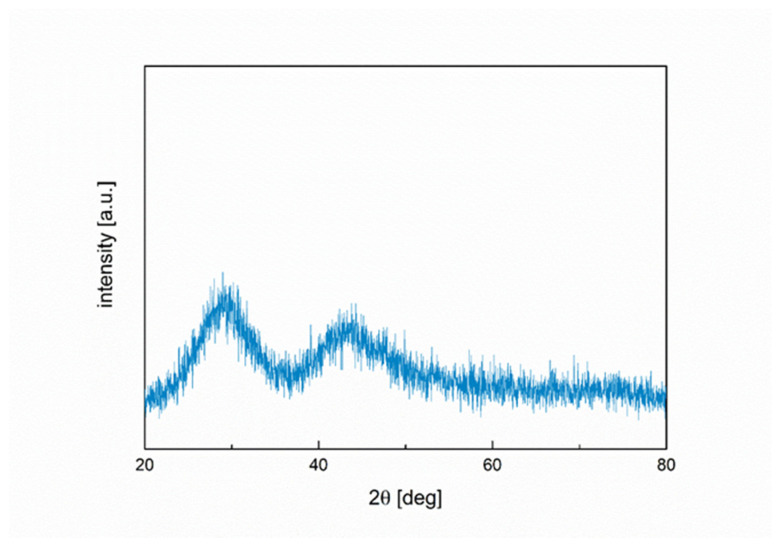
XRD pattern of Schott glass GM31107 powder.

The X-ray diffraction measurement results of Schott glass GM31107 powder are shown in Figure 3.

The diffractogram shows a lack of sharp, well-defined peaks, typical for crystalline phases, and two broad halo maxima, at approx. 29 and 43 deg. This provides evidence that the examined material was ab initio amorphous. At this stage of the study, it should be noted that the matrix of the composite, being glass powder, was amorphous, while the additive in the form of ceramic powder was crystalline. Mixing both materials can contribute to devitrification, which might be desirable considering the mechanical properties of composites.

Table 1 shows the results of high-temperature microscopy observations—characteristic temperatures of the materials: glass GM31107 and the composites marked as K1, K2, and K3 containing 10, 20, and 30 wt.% of YSZ, respectively. Five hundred and forty-five °C is the approximate measured sintering temperature of the glass. Six hundred and twelve °C is the approximate softening temperature. The GM31107 glass displays a hemisphere forming ability at around 716 °C. This point is the most favorable in terms of the wettability of the components of the SOC stack by the glass seal. According to data supplied by the manufacturer, the flowing temperature of the glass is around 840 °C, which means there is quite a wide range for the viscous properties of the material. It is important to remember that the sealing process occurs under mechanical compression, while hot-stage microscopy reveals the behavior of the free-standing sample. The sintering temperature of the composites is around 550 °C, and the level of YSZ content seems to have no impact. This may be explained by no reaction taking place between YSZ and the glass matrix in this temperature range, as the sintering temperature of pure YSZ is much higher (>1400 °C). It can be expected that a further increase in temperature (up to 740 °C) can cause the etching of the YSZ particles and formation of Zr-silicates, which will be discussed in the next section concerning the XRD results. The softening temperature of the composite rises as the YSZ content increases, reaching around 674 °C for the composite with 30 wt.% of YSZ. This agrees well with the results reported recently by Rao and Singh [46] who showed that additions of YSZ (10–30 wt.%) to a silicate glass promoted a gradual increase in dilatometric softening temperature. This effect was discussed in terms of the increased viscosity of the composites as a result of hindering the flow of the glassy matrix by rigid inclusions. A similar trend can be expected for the value of hemisphere temperature; however, the composites with 20 and 30 wt.% of YSZ did not show hemisphere formation in the temperature range of the measurement (up to 1100 °C), and these samples seemed to increase instead of lowering their heights. This is an interesting observation, taking into consideration that the matrix material softens at a much lower temperature. This effect may be due to gas desorption from the samples (as they can absorb water) on heating, combined with the increased viscosity of the composites.

### 3.2. Characterization of the Composites

**Figure 4:**
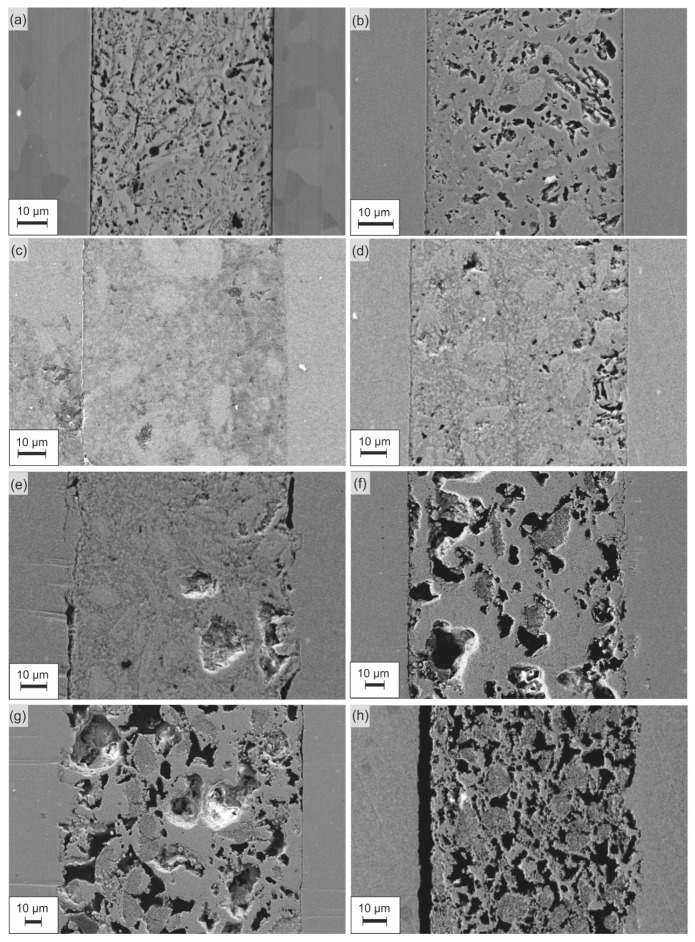
Cross-sections of the obtained samples consisting of 0–70 wt.% YSZ: (**a**) reference glass sample, (**b**) composite with 10 wt.% YSZ, (**c**) composite with 20 wt.% YSZ, (**d**) composite with 30 wt.% YSZ, (**e**) composite with 40 wt.% YSZ, (**f**) composite with 50 wt.% YSZ, (**g**) composite with 60 wt.% YSZ, and (**h**) composite with 70 wt.% YSZ.

**Figure 5:**
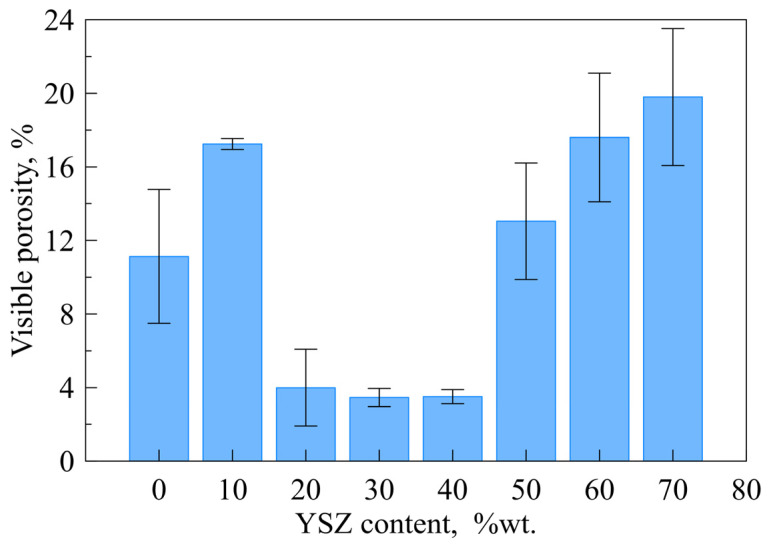
Porosity of the glass and composite layers after the sealing procedure vs. YSZ content.

**Figure 6:**
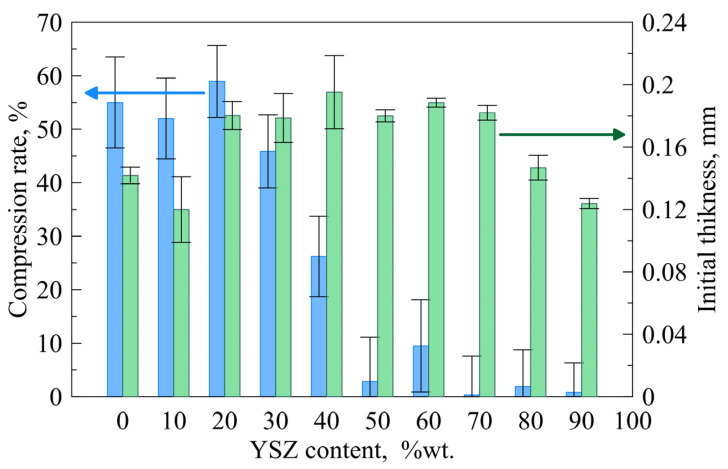
Compression rate and initial thickness of the sample seals after the heating procedure vs. YSZ content.

Heated and polished sandwich samples were observed using a scanning electron microscope. The results of the microstructural analysis for the samples containing up to 70 wt.% YSZ are presented in Figure 4 and Figure 5. Samples containing 80–90 wt.% were visibly not joined with steel after heating, so they were not subjected to this analysis. It can be observed that for composites containing up to 60 wt.% YSZ, the glassy phase remains dense, and the interfaces are well-joined and remain smooth. Only in the case of 70 wt.% YSZ addition was only one side of the composite joined with steel (the bottom one), while the other remained unwetted by the glass matrix. The reference sample and composite containing 10 wt.% YSZ had a considerable number of small pores in the glass matrix. The formation of pores was suppressed by the addition of YSZ in the range 20–40 wt.%, which led to a decrease in porosity down to c.a. 4% (Figure 5). On the contrary, a further increase in the YSZ content resulted in the gradual development of visual porosity, up to 20% for 70% of infill. High porosity and detachment from other surfaces exclude the use of composites with more than 40 wt.% YSZ for sealing applications. A distinct densification behavior of the samples with low YSZ contents (≤10 wt.%) likely originated from the differences in the devitrification process (discussed below) and probably also from the relatively lower initial tape thickness (Figure 6).

Figure 6 shows a comparison of the change in the sample thickness after the heating procedure, which can be interpreted as compression of the gasket. The trend observed is that the compression decreases with the increase of YSZ content in the composite, but this dependence is not linear. The addition of 10–30 wt.% YSZ does not have any significant influence, while the addition of 40 wt.% YSZ reduces the compression by almost half. In the case of gaskets containing YSZ above 50 wt.%, compression of the gaskets during operation is very low, definitely less than 10% of the thickness change. Such phenomena can be useful with regard to obtaining the desired thickness of a seal without using additional support material such as micanite in hybrid seals.

**Figure 7:**
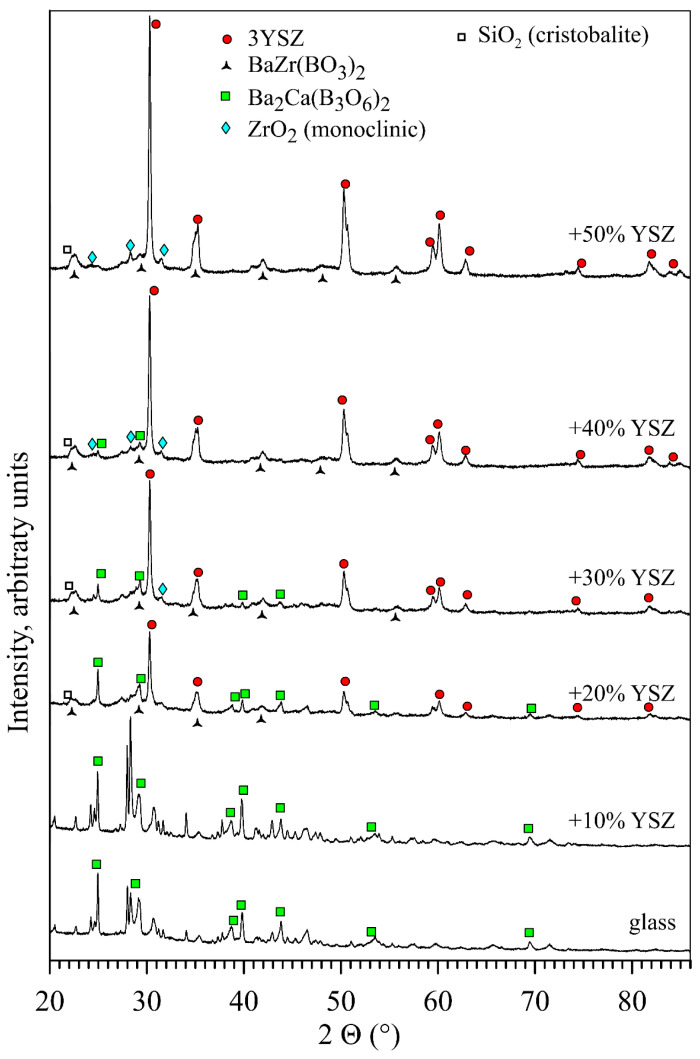
XRD patterns of the glass and composite samples containing 10–50 wt.% YSZ after the thermal treatment cycle.

XRD analysis of the glass and composites samples demonstrates that a noticeable devitrification occurred after the thermal treatment cycle (Figure 7). Importantly, the pattern of devitrification changes when the content of YSZ is higher than 10 wt.%. For the composite with low YSZ content, 10 wt.%, the devitrification of the matrix is similar to that observed for pure glass; some amount of the Ba_2_Ca(B_3_O_6_)_2_ phase (ICDD PDF 04-013-3812) was detected, while other peaks cannot be clearly attributed to the phases expected for the relevant oxide systems or those proposed in the literature for glasses with similar compositions [42,47,48,49,50,51,52,53,54]. It is likely that unidentified reflections in the XRD patterns may originate from metastable complex silicates related to, for instance, celsian BaAl_2_Si_2_O_8_ [55,56], but this cannot be unambiguously concluded from the data. Additions of more than 10 wt.% of the tetragonal YSZ (PDF 01-070-4426) were found to suppress the crystallization of the glass matrix. Although precipitation of the Ba_2_Ca(B_3_O_6_)_2_ phase was still observed for all of the samples, its fraction decreased with increasing YSZ content. Small amounts of several Zr-containing phases were observed in the composites including monoclinic ZrO_2_ (PDF 00-065-0687) and barium zirconium borate BaZr(BO_3_)_2_ (PDF 00-056-0239). Minor segregation of SiO_2_ in the form of cristobalite (PDF 04-008-7833) was also evidenced.

**Figure 8:**
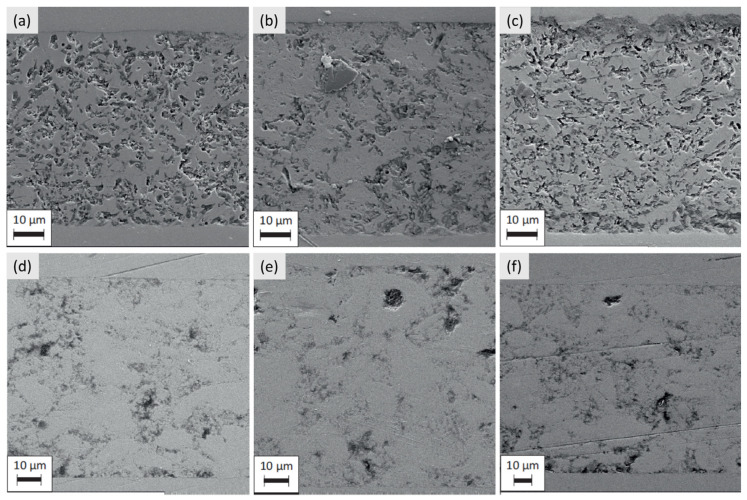
Results of SEM observations of the pure glass and 40 wt.% YSZ composite samples that underwent repeated thermal cycles: (**a**) glass after 3 cycles, (**b**) glass after 5 cycles, (**c**) glass after 10 cycles, (**d**) composite after 3 cycles, (**e**) composite after 5 cycles, (**f**) and composite after 10 cycles.

Taking into account the aforementioned results, the composite with 40 wt.% YSZ addition was chosen for further study to define the stability of the joint undergoing a number of thermal cycles, together with the pure glass as the reference sample. New sandwich samples were prepared and subjected to the heating procedure repeated 3, 5, and 10 times. Figure 8 shows scanning electron microscopy images of the joints machined after the experiment. The junction between the seals and the surface of the steel coupons remains unaffected by repeated heating and cooling processes. There is no sign of delamination or detachment of the seal from the steel surface. What is more, both the pure glass and composite seals show proper wettability of the steel components. The images of the composite suggest that devitrification occurs; however, it does not seem to have a negative influence on the seal.

## 4. Conclusions

In this study, composite glass–ceramic gaskets applicable for SOC stack assembly were prepared, and their properties were studied. The aim of the research was to select a composition of a glass-YSZ composite characterized by low compression, good joining with the surface of ferritic steel interconnectors, and the lowest possible porosity to ensure adequate tightness of the stack compartments. The most favorable combination of properties was found for the composite containing 40 wt.% of yttria-stabilized zirconia. It was characterized by modest compression (c.a. 2 times lower compared to pure glass), low porosity (c.a. 4%), and an excellent junction with Crofer 22 APU^©^. Further study indicated that thermal cycling in conditions approximating the modus operandi of stacks does not impact the quality of a composite seal. It was observed that the addition of more than 10 wt.% of YSZ changed the devitrification mechanism to the formation of Zr-containing phases. The novelty of the work relates to elaborating the guidelines for glass-zirconia composite seals and providing evidence that such a concept can be applied to stacks. This paves the way for the recycling of materials that can serve as additives, thereby reducing the demand for raw materials in the fabrication of SOC stacks.

In conclusion, the results of the experiments conducted during this study confirmed that glass composites reinforced with zirconia powder are promising materials for seals in SOC stacks thus reducing manufacturing costs and improving the mechanical properties of gas-tight junctions.

## Figures and Tables

**Table 1:** High-temperature microscopy of Schott glass GM31107 and composites.

Material	YSZ Content [wt.%]	Sintering Temperature [°C]	Softening Temperature [°C]	Hemisphere Temperature [°C]
Glass GM31107	0	545	612	716
K1	10	550	625	735
K2	20	550	654	-
K3	30	550	674	-

## Data Availability

Data are contained within the article.
